# Next generation sequencing identified two novel mutations in *NIPBL* and a frame shift mutation in *CREBBP* in three Chinese children

**DOI:** 10.1186/s13023-019-1022-8

**Published:** 2019-02-15

**Authors:** Hui Tang, Jing Guo, Siyuan Linpeng, Lingqian Wu

**Affiliations:** Center for Medical Genetics, School of life sciences, Central South University, 110 Xiangya Road, Changsha, Hunan 410078 People’s Republic of China

**Keywords:** Cornelia de Lange syndrome, Rubinstein-Taybi syndrome, *NIPBL*, *CREBBP*, Next generation sequencing

## Abstract

**Background:**

Cornelia de Lange syndrome (CdLS) and Rubinstein-Taybi syndrome (RSTS) are both rare congenital multiple malformation disorders caused by genes associated with transcription. They share a number of similar features clinically. In addition, it is difficult to make a molecular diagnosis rapidly and detect the mosaic mutation when only sanger sequencing is taken. This study aims to report three novel mutations in three Chinese children identified by next generation sequencing.

**Results:**

We describe patient 1 and patient 2 presenting with characteristics of CdLS with mutations in *NIPBL* and patient 3 with a frame shift mutation in *CREBBP* who can be diagnosed as RSTS clinically and also have similar symptoms with CdLS to some extent. The splicing site c.4321-1G > A transversion in *NIPBL* is a mosaic mutation and produces an abnormal transcript bearing the loss of exon 20. The nonsense mutation c.218C > A in *NIPBL* and the frame shift c.1715delC mutation in *CREBBP* generate stop codon and yield the premature termination of proteins.

**Conclusions:**

In general, we detect three novel heterozygous mutations including a splicing mutation and a nonsense mutation in *NIPBL* and a frame shift in *CREBBP*. And several similar features observed in patients indicate the clinical complexity and clinically overlapping of CdLS and RSTS termed “transcriptomopathies”, suggest the underlying molecular mechanism and emphasize the utilization of next generation sequencing technologies.

## Background

Transcriptional regulation of temporospacial gene expression plays an important role in all cellular processes. To achieve this, we need numerous multiprotein complexes to maintain genes in coordinated pattern such as cohesion complex, chromatin remodeling factors [1, 2]. These complexes are composed of conserved core subunits and variant subunits. Destructive mutations in complex protein related with transcriptional regulators bring about a series of disorders which are so-called “transcriptomopathies” and share several clinical features including intellectual disability, growth retardation, characteristic facial dysmorphia and other systemic involvement [3].

In recent years, Cornelia de Lange syndrome(CdLS; OMIM 122470,614,701,610,759,300,590,300,882)is a well-described congenital multiple malformation disorder. The prevalence of CdLS is estimated to be about 1/100,000 birth [4], however the actual figure may be higher because quite a part of patients with milder phenotypes has been confirmed. Five genes are responsible for CdLS: *NIPBL*, *SMC1A, SMC3, RAD21* and *HDAC8*, all encoding constituents of cohesion complex or its associated regulators. Mutations in *NIPBL* account for 60% CdLS probands [5, 6], while approximately 5–10% CdLS patients have mutations in *SMC1A, SMC3, RAD21, or HDAC8* [7–10]. NIPBL is well known for loading the cohesion onto chromatin [11]. Nevertheless, its role in regulating gene transcription also can’t be ignored [12, 13] and its destruction brings about imbalance of multiple genes in patients that prompts the mechanism behind CdLS [14, 15]. Negative results in patients are probably due to somatic mosaicism, new unknown pathogenic genes or overlapping with other diseases. To this day, individuals presenting with CdLS or CdLS-overlapping phenotypes have been reported and detected with causative mutations in chromatin-associated factors like *AFF4*, *ANKRD11*, *KMT2A* and *BRD4* [16–19]. Mutations in transcription repressor ANKRD11 result in KBG syndrome (OMIM 148050), impaired function of histone methyltransferase KMT2A lead to Wiedemann-Steiner syndrome (OMIM 605130) and *AFF4* encoding a core component of the super elongation complex causes a novel syndrome (CHOPS syndrome; OMIM 616368) [16, 20, 21]. *BRD4* encodes a bromodomain protein which binds to cycle-dependent kinase 9 (CDK9) and super-enhancers to regulate transcriptional elongation [22, 23] and may interacts with NIPBL and the core cohesin ring in super-enhancer function [19].

As a congenital multisystemic disorder sharing some similar clinical features with CdLS, Rubinstein-Taybi syndrome (RSTS;OMIM180849,613,684) arises from dysfunction in cyclic-AMP-regulated enhancer binding protein (CREBBP) and E1A binding protein p300 (EP300) as transcriptional co-activators [24, 25]. Moreover, some researchers described a child carrying a novel *EP300* frame shift mutation whose facial features and complex phenotype overlaps CdLS [26].

In this paper, we collect three Chinese patients coming for consultation for the same problem. By next generation sequencing technologies, we identify two de novo mutations in *NIPBL* and 1 bp deletion in *CREBBP* that are never reported before. The child with 1 bp deletion in *CREBBP* can be diagnosed as RSTS. Apparently, there are several similarities between him and CdLS. Our study benefits for understanding the pathogenesis which is indispensable for developing treatments and provide hope for individuals suffering from disease by imbalance of gene transcription.

## Methods

### Patients and controls

This study contains three Chinese children and fully complies with the Tenets of the Declaration of Helsinki and the patient’s parents have written informed consent. We use two cDNAs from normal individuals as controls. Their clinical symptoms are summarized in Table 1.

**Table 1:** Summary of clinical findings and molecular diagnose

	P1	P2	P3
Gender	F	M	F
Gene And Mutations	*NIPBL* c.4321-1G > A	*NIPBL* c.218C > A	*CREBBP* c.1715delC
RefSeq	NM_133433	NM_133433	NM_004380
Origin	De nove	De nove	De nove
Weight at birth	2.85 kg	2.57 kg	U
Age at test	1 years	2 years	9 years
Weight at test	5 kg	7 kg	21 kg
Head circumference at test	40 cm	40 cm	51 cm
Height at test	65 cm	73 cm	110 cm
Growth retardation	Severe	Severe	Light
Intellectual disability	+	+	+
Speech delay	U	+	+
Arched eyebrows/Synophrys	+/+	+/−	+/+
Long eyelashes	+	+	+
Strabismus	–	–	+
Low set ears	+	–	–
Broad or Depressed nasal bridge/Anteverted nostrils	+/+	+/+	+/−
long philtrum	+	+	–
Teeth hypoplasia	U	–	+
Laryngeal cartilage dysplasia	–	+	+
Finger or toe deformity	+	+	+
Hirsutism	–	–	+
Cryptorchidism	–	+	–
Chromosomekaryotype	46,XX	46,XY	46,XX
CNV-seq	–	–	–

*M*: Male, *F*: Female, *U*: Unknown

### DNA extraction and next generation sequencing

Genomic DNA was extracted from peripheral blood samples using the Quickgene DNA Whole Blood Kit L (FUJIFILM, Tokyo, Japan) according to standard extraction methods. A minimum of 3 μg DNA of patient 1 was used to create the DNA libraries and is enriched by the GenCap custom enrichment kit (MyGenostics Inc., Beijing, China) based on the design of a custom-made gene panel including the known CdLS genes and patient 2’s DNA was under next generation sequencing for 4000 genes panel (Mega Genomics Inc., Beijing, China). And 2 μl peripheral blood of patient 3 was needed for the whole-exome sequencing (Berry Genomics Inc., Beijing, China).

### Mutation analysis by direct sequencing

Then we filtrated and analyzed the data. Two mutations in *NIPBL* and one 1 bp deletion mutation in *CREBBP* came into sight. The mutations were validated by sanger sequencing. All necessary regions of *NIPBL* and *CREBBP* were amplified by the polymerase chain reaction (PCR). The PCR primers were designed using Primer5 software (Premier Biosoft International, Palo Alto, CA). Three pairs of primers were designed (Table 2).

**Table 2:** Primers for PCR amplication

Primer name	F, 5′ → 3′	R, 5′ → 3′
NIPBL-EXON20	CATTTTCATTCTAAATGGCAGGT	TGACGGTTCAATATAATGGTGGT
NIPBL-EXON3	TTGTTAGGAAGAGGAGGAATGC	ATTCTCCCTCGGATATGGAT
CREBBP-EXON8	CTCAGGAGCGATACTAATGAAGCAG	CCGAGGACATAGAGTGTGGGAAT

### RNA extraction and identification of splice transcripts by RT-PCR

Total RNA from blood leukocytes of patients and controls was extracted using a standard Trizol method. cDNAs were synthesized from RNA using RevertAid First Strand cDNA Synthesis Kit (Thermo, USA). Exons 19–22 in *NIPBL* of patient 1 were amplified with primers RTF (5′- TGTGACATTGTTAGCAGCTTATCAG-3′) and RTR (5′- CTGTTCGCATAGCTGTTTCATAAGA-3′). The normal length of the products is 442 bp. The products were verified by polyacrylamide gel electrophoresis (PAGE) and were sequenced by sanger sequencing (Biosune, China).

### Pyrosequencing

The following pyrosequencing primers were designed to amplify a 108 bp *NIPBL*exon20: NIPBL-F 5′- TGATATTATTTTTTGGTTTGTTTTC-3′ and NIPBL-BR.

5’-Biotin-CTTCCAAAATTAACTGCCTATGT-3′. Then the product was checked on a 1% agarose gel and was pyrosequenced on a PyroMark Q96 ID (Qiagen, Hilden, Germany) using the sequencing primer 5’-AACTTGGAATCTTATAATTACTAAAC -3′. The data was analyzed by PyroMark CpG software 1.0.11.

## Result

### Clinical features

**Fig. 1:**
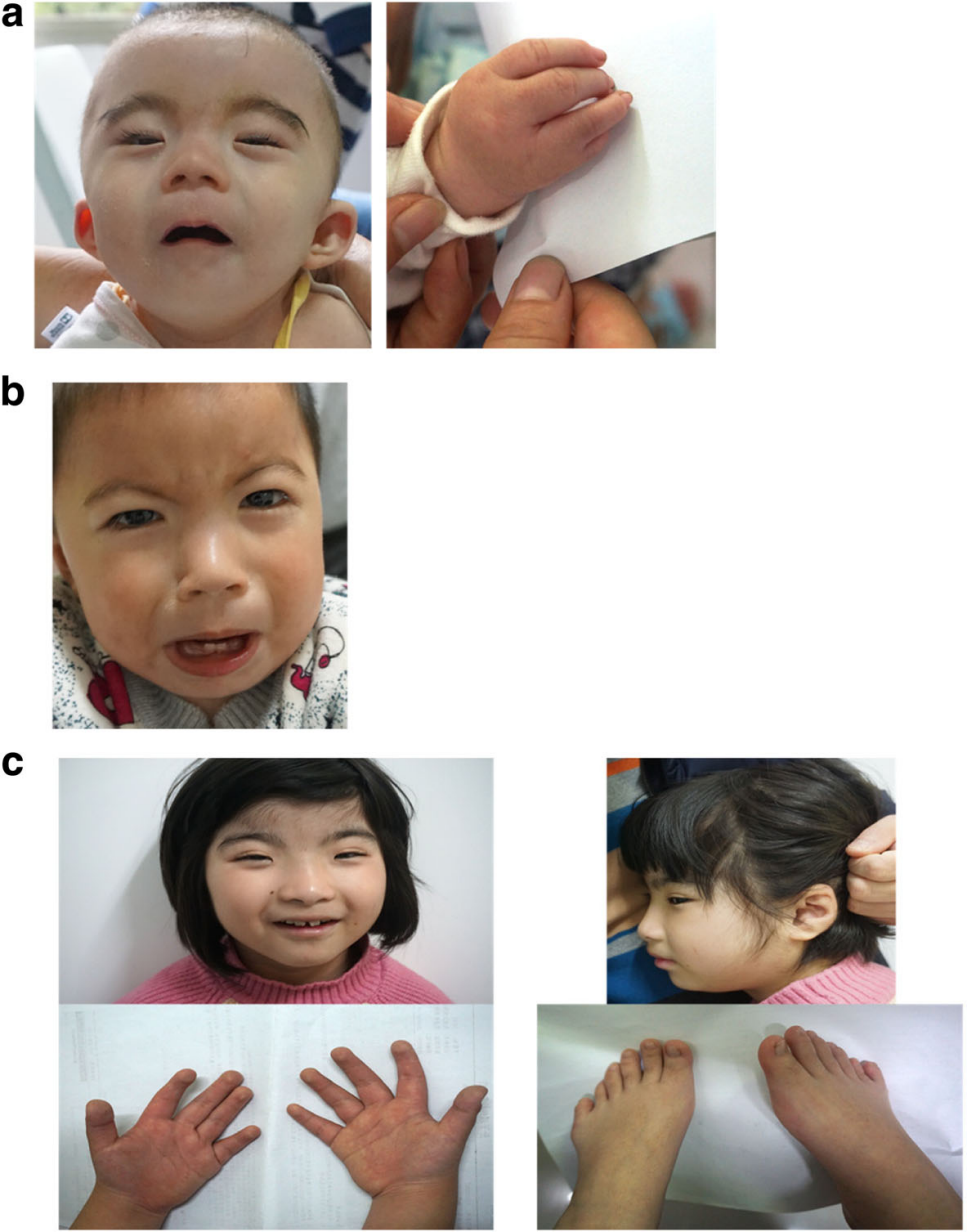
Facial features and limb anomalies of all the patients: **a** Facial features and hand defects of patient 1. **b** Craniofacial anomalies of patient 2. **c** Facial malformation, profile, fingertip pads in hands and polydactyly of toes in patient 3

Patient 1 (P1) was a 1-year-old girl who was born at 38 weeks of gestation. Birth weight was 2720 g (− 1.3 SD), length 41 cm (− 5 SD), and head circumference was 33 cm (− 0.8 SD). During the period of neonatal evaluation, she exhibited multiple malformations, namely arched eyebrows, long eyelashes, long philtrum, orbital hypertelorism, droop eyelid, low-set ears, depressed nasal bridge, anteverted nostrils, high palate, posterior hairline, short neck, small hands with abnormal palmar crease, curved 5th finger and partial webbing between 4th and 5th fingers (Fig. 1a). And an ultrasound inspection at 32 weeks also revealed intrauterine growth restriction, tricuspid regurgitation and enlarged foramen ovale. At 14 months, her weight was 5000 g (− 5 SD), length 65 cm (− 5 SD), and head circumference was 40 cm (− 4 SD) which showed the patient had serious growth retardation. And the child died at 2 years old. Until that, she could not sit alone, walk alone and speak. Taken together, she received a clinical diagnosis of CdLS.

Patient 2 (P2) was a 2-year-old boy whose birth weight was 2570 g (-2SD) and head circumference was 30 cm (-3SD). His craniofacial features included arched eyebrows, long eyelashes, broad and depressed nasal bridge, anteverted nostrils and teeth hypoplasia (Fig. 1b). He had small hands with bilateral transverse palmar crease. He also developed laryngeal cartilage dysplasia and hypoplastic male genitalia. MRI showed slightly widening of frontotemporal extracerebral space. When coming for genetic counseling, he was 2 years old and his weight was 7000 g (−5SD), length 73 cm (−5SD), and head circumference was 40 cm (−5SD). According to his abnormal phenotypes, a mild kind of CdLS was consistent.

Patient 3 (P3) was a 9-year-old girl whose weight was 21,000 g, length 110 cm, and head circumference was 51 cm. At the age of 6 years, her weight was 15,000 g (−2SD), length 98 cm (−5SD), and head circumference was 48 cm (−2SD). She was evaluated for severe speech delay, light growth retardation, low anterior hairline, hypoplastic maxilla, heavy and arched eyebrows, synophrys, long eyelashes, iris hypoplasia, strabismus, broad nasal bridge, widely spaced incisors, an atypical smile, persistent fingertip pads, polydactyly of toes, hairy back and forehead and laryngeal cartilage dysplasia (Fig. 1c). Her great toes, broad terminal phalanges and grimacing smile as characteristics of RSTS seemed support the diagnosis of it. Nevertheless, we did not observe a beaked nose in her profile that can be seen in a majority of patients (Fig. 1c). Her polydactyly is also rare in RSTS but is common in CdLS. And some features like low anterior hairline, hypoplastic maxilla, heavy and arched eyebrows, synophrys, long eyelashes indeed overlapped with CdLS. The chromosome karyotype and CNV-seq result were normal.

### Identification of mutation by next generation sequencing and sanger sequencing

**Fig. 2:**
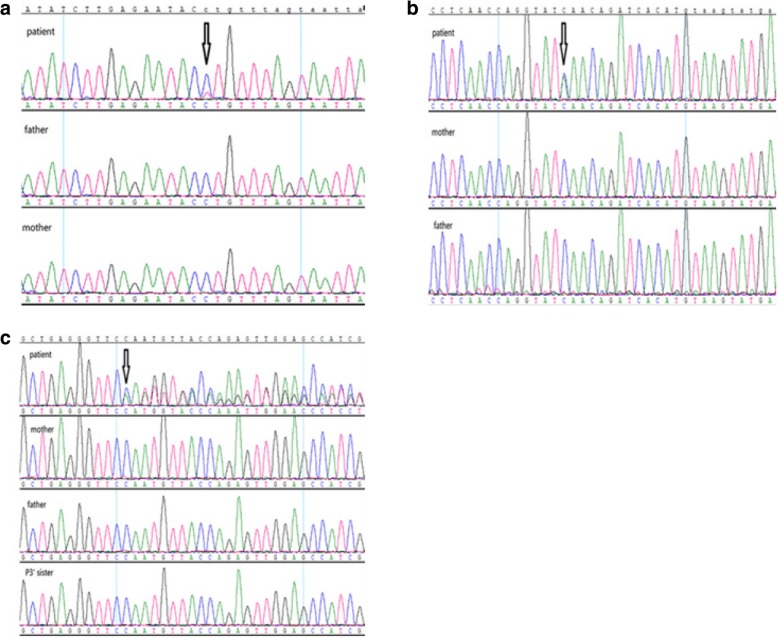
Genetic characterization of the families with CdLS: **a** A heterozygous mosaic mutation (c.4321-1G > A) in *NIPBL* was found in p1. Her parents were absent from this mutation. **b** A heterozygous mutation (c.218C > A) in *NIPBL* was found in p2. His parents did not show this mutation. c A heterozygous deletion (c.1715delC) in *CREBBP* was found in p3 and was not detected in her parent and her health sister

The sequencing depth and the coverage of all dates met the qualification for subsequent analysis. After filtering the variants by the minor allele frequency (MAF) < 0.01 in the 1000 Genome Project and The Exome Aggregation Consortium (ExAC), referring to the phenotypes and using some online websites to predict the pathogenic possibility and regions conservation such as SIFT (http://provean.jcvi.org/index.php), PolyPhen-2 (http://genetics.bwh.harvard.edu/pph2/), MutationTaster (http://www.mutationtaster.org/) and HSF (http://umd.be/HSF3/), we founded a mosaic de novo heterozygous mutation c.4321-1G > A (p.F1442Kfs*3) in *NIPBL* (NM_133433) in P1 with 29% mutation ratio, a de novo heterozygous mutation c.218C > A (p.S73*) in *NIPBL* (NM_133433) in P2 and a de novo heterozygous 1 bp deletion c.1715delC (p.G572Efs*17) in *CREBBP* (NM_004380) in P3. All mutations have not been reported in Human Gene Mutation Database (HGMD), dbSNP138 or Leiden Open Variation Database (LOVD) and were confirmed by sanger sequencing (Fig. 2). Meanwhile, the sequence result of c.4321-1G > A in *NIPBL* revealed a mosaicism due to the low signals.

### RNA analysis and pyrosequencing

**Fig. 3:**
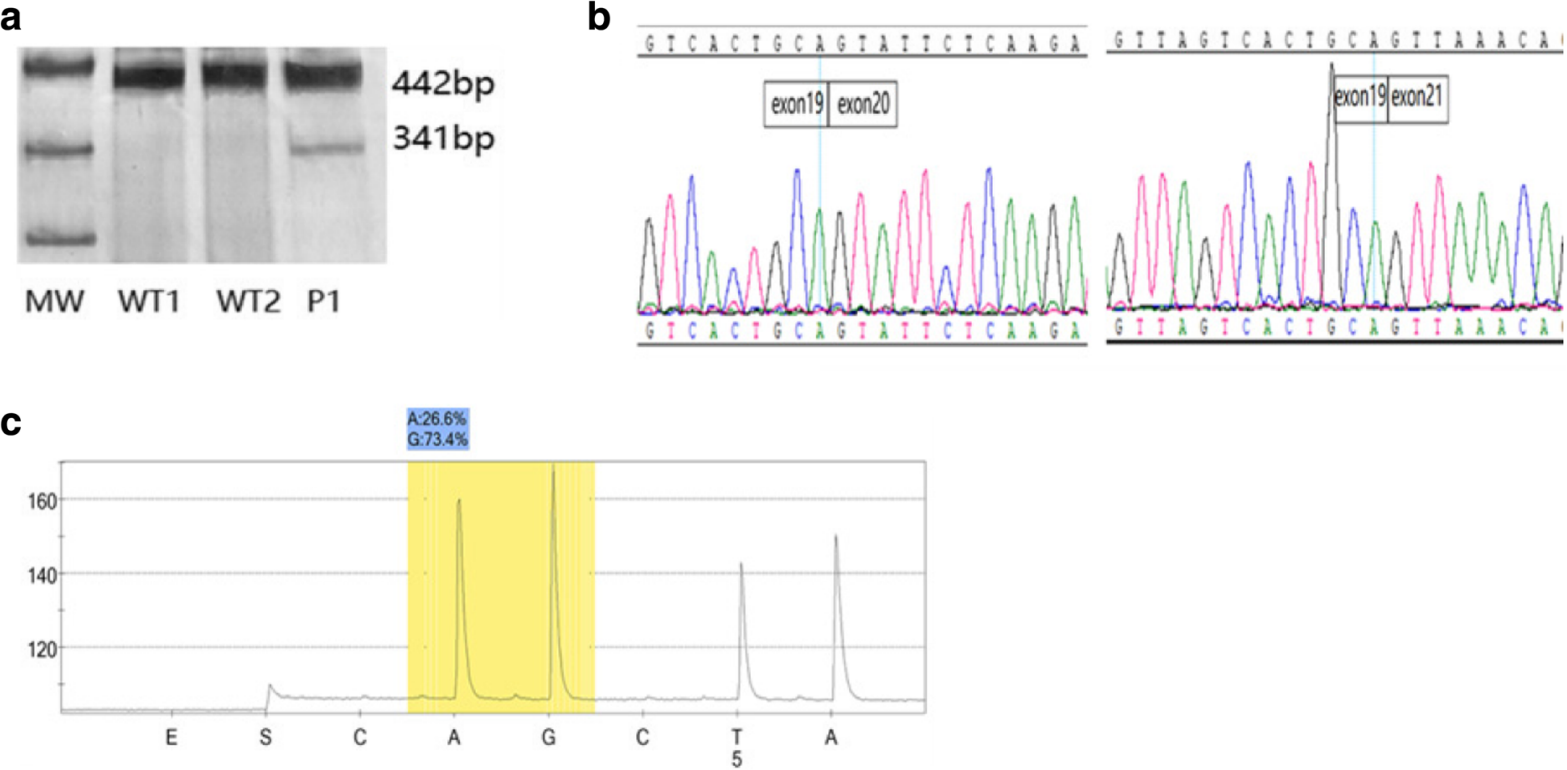
Exon-trapping analysis and sequencing: **a** Polyacrylamide gel showed RT-PCR products. WT1 and WT2 exhibited one normal splicing RT-PCR product (442 bp); P1 exhibited a normal product of 442 bp and an additional band of 341 bp. **b** Sanger sequencing testified a loss of exon 20 of abnormal product compared to normal product of 442 bp. **c** The mosaicism level of NIPBL c.4321-1G > A was detected by pyrosequencing: the mutated allele 26.6% and the wild allele 73.4%

Two cDNAs from normal individuals were used as controls and only a 442 bp wild type product was detected. Nevertheless, for P1, there were two PCR products, a 341 bp aberrant transcript and a 442 bp wild type product, respectively (Fig. 3a). Sequencing results of the abnormal product displayed a loss of a 101 bp fragment representing the entire exon 20 of *NIPBL* (Fig. 3b).We used pyrosequencing to quantify the fraction of the mutated allele from p1’ peripheral blood. The frequency of the mutated allele was 26.6% and the wild allele was 73.4% (Fig. 3c).

## Discussion

Here, we described and characterized two novel mutations in *NIPBL* and a de novo mutation in *CREBBP* using targeted exome sequencing from patient’s peripheral blood lymphocytes.

P1 with the A > G transition locating at c.4321 in *NIPBL* had typical facial abnormity of CdLS and bore light malformation in hands performing as curved 5th finger and partial webbing between 4th and 5th fingers. Her abnormal degree fall in the moderate group and was in accordance with a previous report that patients with splicing mutations got the score from mild to moderate [27, 28]. The mutated allele c.4321-1G > A was present in 14 of 48 (29%) reads in exome sequencing and the mutation-related signal was smaller than that of the wild-type allele in sanger sequencing which suggested that it is a mosaic mutation. And the results of subsequent pyrosequencing using peripheral blood lymphocytes could confirm the mosaicism of this mutation because mutant type accounted for 26% which suggested next generation sequencing contributed to detect mosaic mutation. Unfortunately, the child died at 2 years old so that we didn’t get the samples of other tissues. It was worth noting that P1 performed worsening of weight from − 1.3SD at birth to -5SD at 14 months of age. We speculated her severe growth retardation resulted from gastroesophageal reflux presenting in more than 95% of patients with typical CdLS. In view of her deterioration clinical chart, aspiration/reflux and pneumonias as the complications of the gastroesophageal reflux may explain the premature death. And respiratory causes including aspiration/reflux and pneumonias are the most common primary causes in CdLS [29]. The mutation yielded an exon 20 deletion transcript and a normal one. Another study found that the mutation c.4321G > T (p.F1442KfsX3/p.V1441 L) in *NIPBL* also produced two splicing transcripts, one losing exon 20 and another transcript possessing p.V1441 L protein [30]. Compared with this, the splicing mutation c.4321-1G > A may give rise to more severe phenotypes because individuals with missense mutations tended to have milder phenotypes [6]. In conformity with that, our patient presented with webbing between 4th and 5th fingers in limb malformations and tricuspid regurgitation and enlarged foramen ovale in cardiovascular abnormality that were absent in the patient with c.4321G > T. But c. 4320 + 5G > A and c.4320 + 2 T > A produced the same aberrant transcript with the deletion of exon 19 (p.V1414_A1440del) and caused severe limb malformation [29, 30]. It is in contradiction that people with splicing mutations disrupting the reading frame is associated with the more severe phenotypes [31]. These suggest the region of exon 19 may play a vital role in limb development.

Simultaneously, we observed that the mutation c.218C > A (p.S73*) in P2 leaded to milder phenotypes and it was inconsistent with genotype-phenotype correlation that the nonsense mutations were associated with a more severe phenotype [32]. Former research demonstrated that the expression reduction to 70% of NIPBL could generate multiorgan defects like disruption in gut and heart development and the decrease to 50% was lethal [32, 33]. Hence, cells are very sensitive to the dosage of NIPBL. And the NIPBL expression levels in probands may conduce to predicting the phenotypic severity [34, 35]. The finding that phenotypes associated with p.R827GfsX2 in seven people arranged from mild to severe in limb defects indicates the influence of modifiers in CdLS [30]. To this patient, we surmise that other factors such as underlying genetic modifiers affect the deregulation of NIPBL resulting in his mild performance. There is no denying that we need more patients to confirm the genotype-phenotype correlation. To sum it up, these findings suggest the severity of phenotypes in CdLS is not merely determined by the type of mutations and other factors can also influence it.

Besides two novel variants in *NIPBL*, a frame shift variant c.1715delC in *CREBBP* was also obtained. *CREBBP* is one of pathogenic genes of Rubinstein-Taybi syndrome. The gene encodes cyclic-AMP-regulated enhancer binding protein CBP which is a coactivator protein of CREB (cAMP-response element-binding protein) acting as transcription factors and works in transcription by modulating chromatin and helping RNA polymerization [36]. Another causative gene of RSTS is *EP300* [25]. RSTS is one of the first multiple malformation syndromes because of dysregulation of gene expression [24]. *CREBBP* mutations are detected in 50–60% of RSTS patients while only 3–8% carry an *EP300* mutation [25, 36]. A former research described a patient with features extremely similar to CdLS possessing a *EP300* mutation [26]. On account of P3’ phenotypes, she was likely more aligned with RSTS. But she didn’t have a beaked nose. Her polydactyly and a fraction of facial features indeed confused us. P3 shared several similarities with CdLS, including intellectual disability, growth retardation, speech delay, anterior hairline, hirsutism, droop eyelid, hypoplastic maxilla, arched eyebrows, long eyelashes. It suggests the underlying common mechanism of CdLS and RSTS. People find CREBBP and EP300 can regulate the expression of its targeted gene [37, 38] and EP300 is recruited at enhancers [39, 40]. And as early as 1999, Rollins et al. had described NIPBL facilitated enhancer-promoter communication and activated gene expression [12]. Therefore the role of CREBBP, EP300 and NIPBL in gene transcription, chromatin remodeling and recruitment to enhancers provide clues to the intersection of potential molecular mechanism and clinical features between CdLS and RSTS.

## Conclusions

In summary, we found two deleterious mutations in *NIPBL* and a frame shift mutation in *CREBBP*. Interestingly, the patient with the mosaic splicing variant carried moderate phenotypes while the nonsense variant resulted in a mild form. And the child with mutation in *CREBBP* got a primary clinical diagnosis of RSTS and the molecular results could confirm it, although we still observed similar phenotypes between P3 and CdLS. Our findings demonstrated the clinical complexity and similarities of CdLS and RSTS and supported previous study that pathogenic gene participating in the same pathway could lead to similar clinical malformation. Furthermore, we recommended the utilizing of next generation sequencing containing causative genes of “transcriptomopathies”. It’s helpful to detect the somatic mosaicism as well. The research emphasizes the significance of making accurate diagnoses for CdLS and understanding the molecular mechanism among the “transcriptomopathies” which may make it possible for finding cure therapies.

## Abbreviations

CBP: Cyclic-AMP-regulated enhancer binding protein
CDK9: Cycle-dependent kinase 9
CdLS: Cornelia de Lange syndrome
CREB: cAMP-response element-binding protein
ExAC: Exome Aggregation Consortium
HGMD: Human Gene Mutation Database
LOVD: Leiden Open Variation Database
MAF: Minor allele frequency
p300: E1A binding protein p300
PAGE: Polyacrylamide gel electrophoresis
PCR: Polymerase chain reaction
RSTS: Rubinstein-Taybi syndrome
